# Glycosylation and its research progress in endometrial cancer

**DOI:** 10.1007/s12094-022-02858-z

**Published:** 2022-06-25

**Authors:** Congli Pu, Biyuan, Kai Xu, Yingchao Zhao

**Affiliations:** 1 Cancer Center, Union Hospital, Tongji Medical College, Huazhong University of Science and Technology, Wuhan, 430030 China; 2 Department of Otorhinolaryngology Head and Neck Surgery, Tongji Hospital, Tongji Medical College, Huazhong University of Science and Technology, Wuhan, 430030 China; 3 Institute of Radiation Oncology, Union Hospital, Tongji Medical College, Huazhong University of Science and Technology, Wuhan, 430030 China

**Keywords:** Glycosylation, Endometrial cancer, Biomarkers, Immunotherapy

## Abstract

Endometrial cancer (EC) is one of the most common tumors in the female reproductive system, which seriously threatens women's health, particularly in developed countries. 13% of the patients with EC have a poor prognosis due to recurrence and metastasis. Therefore, identifying good predictive biomarkers and therapeutic targets is critical to enable the early detection of metastasis and improve the prognosis. For decades, extensive studies had focused on glycans and glycoproteins in the progression of cancer. The types of glycans that are covalently attached to the polypeptide backbone, usually via nitrogen or oxygen linkages, are known as N‑glycans or O‑glycans, respectively. The degree of protein glycosylation and the aberrant changes in the carbohydrate structures have been implicated in the extent of tumorigenesis and reported to play a critical role in regulating tumor invasion, metabolism, and immunity. This review summarizes the essential biological role of glycosylation in EC, with a focus on the recent advances in glycomics and glycosylation markers, highlighting their implications in the diagnosis and treatment of EC.

## Introduction

According to the global incidence and mortality of endometrial cancer (EC), regions, such as North America and Europe, rank at the front of global [1]. In the United States, endometrial cancer has been identified as the third most common cancer in women aged between 20 and 39, with annual incidence rates of about 15 to 25 per 100,000 women [2]. Approximately, 65,950 new cases of EC and 12,550 related deaths have been reported in 2021 [3]. But South Africa and several countries in Asia showing the largest increase, such as Japan, the Philippines, and Singapore[4], the age-standardized incidence rate ranges between 5.5 per 100,000 in Central Asia and 70.9 per 100,000 in East Asia. The age-standardized mortality rate ranges between 3.2 per 100,000 in Central Asia and 1.9 per 100,000 in East Asia, even though the death rate in East Asia is the most significant globally [5]. The five-year survival rate for EC patients following appropriate therapy is 80% [6], but the median survival time for stage III–IV EC patients is 9 to 10 months [7]. Patients with higher-stage EC are more likely to suffer from recurrence and mortality, making its prevention increasingly challenging [8]. Therefore, early diagnosis and prediction of prognosis for patients with EC are critical for improving women’s health globally.

Among the various post-translational modifications of proteins, glycosylation is a very important one, which directly interacts with the surroundings or indirectly changes the conformation, stability, and turnover of the proteins [9]. Glycoproteins are widely distributed, including membrane receptors, adhesion molecules, extracellular matrix proteins, intracellular kinases, and transcription factors [10]. With a deeper understanding of glycosylation and the continuous development of mass spectrometry (MS) technology, accumulating data implicates the indispensable role of protein glycosylation in health and disease [11]. High-throughput glycoproteomics technologies have enabled the analysis of thousands of proteins N-glycans in ovarian cancer (OC) [12], providing a platform for the study of glycosylation in EC. However, compared to other tumors, limited studies have investigated the role of protein glycosylation in EC. In this review, we focused on the recent advances in the literature related to glycosylation and glycoproteomics, to better illustrate their roles in the pathogenesis of EC, aiming to identify new tumor-associated glycosylated biomarkers and their clinical applications.

## Overview of glycosylation

### Definition of glycosylation

In eukaryotes, the vast majority of protein glycosylation in the cell occurs along the secretory pathway, under the regulation of glycosyltransferases and glycosidases. The carbohydrates are transferred to the amino acid residue on the protein forming a glycosidic bond. The initial synthesis of the peptide chain of the glycoprotein occurs in the ribosome, and most glycoproteins need to enter the endoplasmic reticulum for modification and folding, such as N‑glycans modification, while for the O-glycans, they need to enter the Golgi apparatus [13]. According to the nitrogen or oxygen linkages attached to the polypeptide backbone, the glycoproteins are usually defined as N‑glycans or O‑glycans, respectively. N-glycosylation refers to the amino acid residues of the asparagine side chain in a polypeptide chain that are connected to *N*-acetylglucosamine (GlcNAc) of the N-glycan chain [14]. While O-glycosylation is a type of glycosylation wherein a carbohydrate group forms an O-glycosidic bond with the hydroxyl group of an amino acid side chain in a peptide chain. The hydroxyl groups that can be used for bonding are mainly the alcoholic hydroxyl groups of serine and threonine, but in some instances, the hydroxyl groups of hydroxylysine and the phenolic hydroxyl group of tyrosine may also be involved. After N-glycosylation or O-glycosylation, a series of fucosylation and sialylation are required to complete the assembly. The addition of sialic acid or fucose moieties to the N-linked or O-linked glycoproteins is one of the most frequently occurring modifications in cancer [15]. The glycosidic bond is different from the above, such as in C-glycosylation, wherein the mannose is linked to the tryptophan through the carbon–carbon bond [16]. If the glycosidic bond modification site is cysteine, it is called S-glycosylation [17]. P-glycosylation involves the attachment of phosphorylated glycans to a serine or threonine and is only observed in lower eukaryotes [18].

Other major classes of glycoconjugates include proteoglycans and glycosphingolipids. Proteoglycan is a protein with a large number of glycosylation modifications and is an important component of the extracellular matrix. Proteoglycans are formed by the covalent attachment of the core protein to one or more glycosaminoglycan chains, and their carbohydrate content is usually higher than that of general glycoproteins. Glycosylphosphatidylinositol is a complex glycolipid composed of mannose, glucosamine, phosphoethanolamine, and inositol phospholipids, which can be covalently linked to the carboxyl terminus of some proteins, anchoring them to the cell membranes for a variety of biological functions [19].

**Fig. 1:**
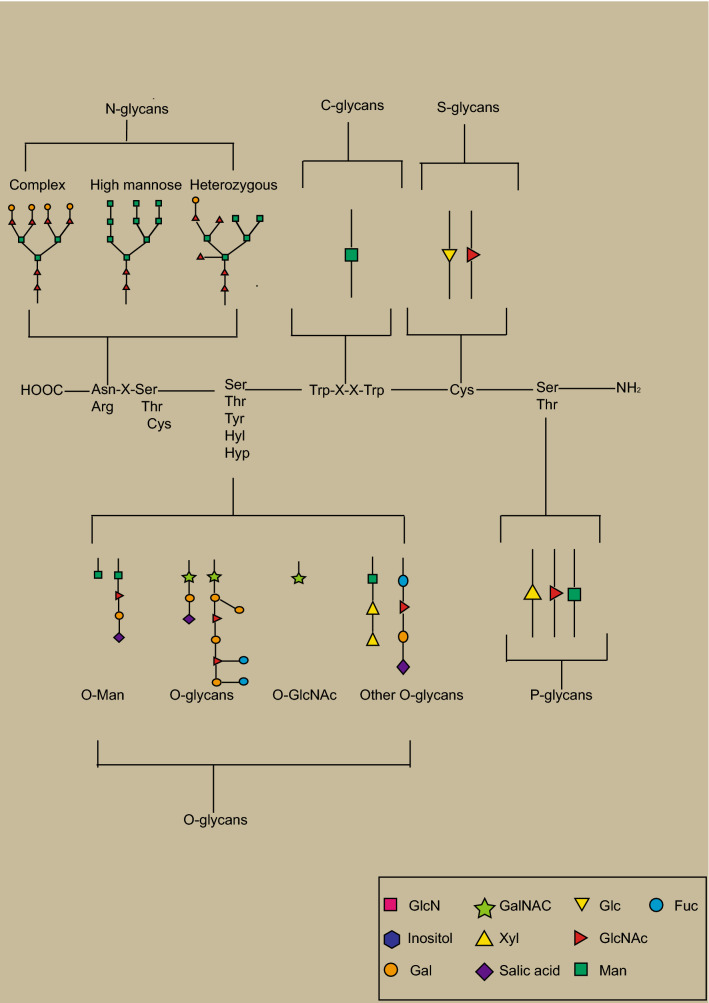
The types of glycosylation

In general, glycans have important biological functions due to their high proportion and wide distribution in cells [19, 20]. Figure 1 shows the synthetic routes for the different types of glycosylation.

### Glycosylation changes in cancer and normal cells

Glycosylated proteins participate in various biological processes in the cell. Aberrant glycosylation is closely related to many pathological processes, such as tumorigenesis and inflammatory response [21]. Meanwhile, due to the complexity of glycosylation and substrate binding sites and the diversity of the structure of the carbohydrate chain itself, glycan modifications are protein-specific, site-specific, and cell-specific [22]. In general, several modifications are observed in the glycosylation pathway that occurs in cancer cells, including the aberrant expression of glycoproteins or glycosyl compounds, alterations in the sites and structures where glycans are linked to the amino acids, abnormal localization and expression of the corresponding glycosyltransferases and glycosidases during glycan synthesis, and somatic mutations [23, 24].

The most frequent changes in glycosylation in cancer are the abnormal sialylation and fucosylation, O-glycan truncation, and N/O-linked glycan branching. Altered sialylation and fucosylation are closely associated with the development and progression of cancer, with the altered sialyltransferase expression leading to the formation of specific sialylated structures [25]. Similar to sialylation modifications, the process of fucosylation relies on a series of fucosyltransferases (FUT1-11). Fucosylation is further divided into two categories, including terminal fucosylation, and core fucosylation. Fucosyltransferase 8 (FTU8) is the most important FTU in mammalian cells, which catalyzes the transfer of GDP-β-L-fucose to the N-sugar chain of Asn in the adjacent *N*-acetylglucosamine (GlcNAc) residues to form core fucose [26]. Altered expression of polypeptide GalNAc transferases results in the incomplete synthesis of O-glycans, known as truncated-O-glycans, and is observed in about 80% of cancers [27]. The disaccharide Thomsen–Friedenreich antigen (T antigen) and the monosaccharide GalNAc (Tn) and their sialylated forms (sT and sTn) are some of the truncated glycans [28]. What’s more, the frequently occurring N/O-linked glycan branching changes in cancer cells cause the overexpression of complex β1,6-branched N-glycans, as a result of the increased activity of β1,6 N-acetylglucosaminyltransferase V, which is regulated by the RAS/RAF/MAPK signaling pathway in cancer [29].

### Research techniques related to glycosylation in gynecological oncology

Proteins may contain multiple glycan modification sites. The type of carbohydrate and occupancy rates at each site may be different, while a specific site may also contain multiple types of glycan structures [30]. Due to the complexity of glycosylation modifications, conventional experimental methods for gene and protein measurements, such as ELISA [31], immunohistochemistry (IHC) [32], and polymerase chain reaction (PCR) [33], are insufficient. The advances in MS-based methods have led to a gradual increase in glycosylation research in recent years, providing an effective and versatile tool for glycan and protein analysis [34]. The current research on glycoproteins is mainly based on three methods: the intact glycoproteins/glycopeptides; the glycopeptide after the glycoprotein is digested by enzyme; and the structure of the glycans released by chemical method or enzyme cleavage method [35]. Before subjecting the samples to MS, some of them undergo release, separation, and enrichment for glycans [36]. Some reviews have specifically described the technical approaches for preparing such samples [37, 38]. Due to the low abundance of glycoproteins and glycosylated peptides in the biological samples, a series of enrichment analyses are conducted before the analysis, such as lectin enrichment and hydrophilic affinity enrichment [39]. Commonly used techniques for characterizing glycans structure include capillary electrophoresis, high-performance liquid chromatography and MS technology, especially the matrix-assisted laser desorption ionization-time-of-flight mass spectrometry (MALDI-TOF-MS), and electrospray ionization mass spectrometry (ESI/MS) [37, 40]. Although these methods can qualitatively and quantitatively assess the structure of glycosyl and glycopeptides, they lack information regarding the binding sites of glycans and glycopeptides. Therefore, the analysis of intact glycopeptides is more suitable [41]. The widely used tandem MS peptide fragmentation modes include collision-induced dissociation [42], high-energy-induced dissociation, and electron transfer dissociation [43]. The latest updates on the methodology used to detect glycosylation changes in gynecologic oncology were summarized and listed in Table 1.

**Table 1:** Glycosylation research techniques in gynecologic oncology

Sample	Description	Analysis methods	Reference
Serum	CA153 and MUC1	ELISA	[31]
	94 EC patients and 112 healthy control	HILIC-UPLC chromatogram	[44]
	3 serum banks	Lectin-based ELISA assay and Quantitative MS	[45]
	146 EC patients (stage I, 98; stage II, 15; stage III, 17; stage IV, 16)	ELISA	[33]
	healthy women and stage recurrent OC	MALDI-TOF/TOF	[46, 47]
Tissue	28 EC FFPE slides	MALDI-TOF/MSI	[48]
	CJ2 human OC tissue array	IHC	[49]
	78 OC tissues	IHC	[50]
	Normal controls (*N* = 24) and malignant serous OC (*N* = 24)	PCR	[51]
	Normal (*N* = 18) and malignant (*N * = 20) endometrium	Immuno- and Lectin-histochemical	[52]
Urine	Endometrial, Ovarian, and Cervical Cancer	CMB lectin immobilized PS10 chip and SELDI-TOF	[53]
	postmenopausal women with OC and benign conditions	SELDI-TOF–MS	[54]
	Stages IB and IIA/B EC (*N* = 7), Control urine samples (*N* = 11)	MALDI-TOF /TOF	[55]
Cell	HEC-1B cells culture medium	MALDI-TOF/TOF mass spectrometer	[56]
	OC Cell Supernatants	HILIC-UPLC	[57]
	Cervical cell lysates	PCR	[58, 59]
	EC cell lysates	ELISA	[60]
Ascites	183 OC metastasis ascites	HPLC–Chip/TOFMS	[61]
	18 EOC patients and the serum of 20 age-matched controls	MALDI-TOF–MS	[62]
	Benign ovarian cyst (*N* = 10) and peritoneal effusion (*N* = 20) fluid	Electrospray ionization-LTQ Orbitrap tandem mass spectrometry	[63]
Extracellular vesicles	Extracellular vesicles from ovarian carcinoma cells	MALDI-TOF/TOF	[64]

## Role of glycosylation in endometrial cancer

**Fig. 2:**
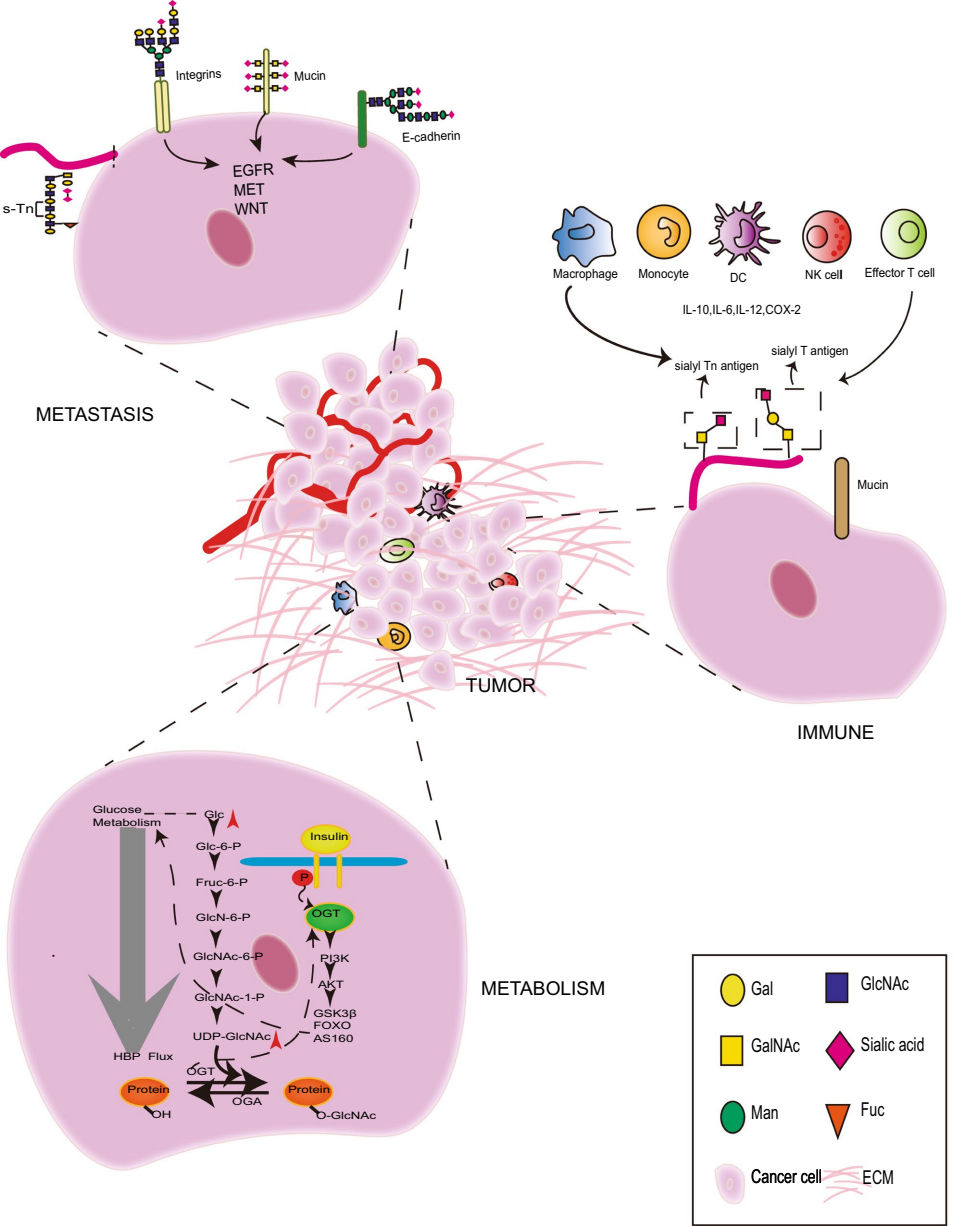
Role of glycosylation in the development and progress of endometrial cancer

Glycans can alter protein conformation and structure, thereby modulating the functional activity of the protein. In this part, we discuss specific examples to highlight the diverse roles of glycosylation in EC. We also try to unravel the biological significance of glycan-based interactions to decipher the molecular mechanisms of tumorigenesis. The role of glycosylation in EC is presented in Fig. 2.

### Glycosylation in tumor invasion and migration

Tumor invasion and metastasis are usually closely associated with the extracellular matrix [65]. Glycoproteins, glycolipids, and glucosamine are important components of the cell surface. The complex carbohydrates attached to the membrane proteins and extracellular matrix proteins, such as E-cadherin [66], integrins [67], Mucin1 (MUC1) [68], and CD44 [69], alter the structure and function of the glycoproteins, as well as intracellular signaling, to promote tumor metastasis. E-cadherin is a widely expressed transmembrane glycoprotein in cancer and is a specific indicator of the loss of epithelial integrity. The reduced expression of E-cadherin in EC associated with deep myometrial invasion and poor differentiation [70]. Kurita et al. showed that positive staining for GalNAc transferase 6 (GalNAc-T6) was significantly associated with positive staining of E-cadherin and advanced grade of EC. Furthermore, the overexpression of GalNAc-T6 enhanced the ability of cell–cell adhesion and the characteristic differentiation found in the early phase of EC invasion [71]. MUC1 is a transmembrane glycoprotein whose glycosylation is altered in the malignant cells, owing to the extracellular heavily glycosylated domain. It has been reported that glycosylation-modified MUC1 promotes tumor growth by regulating the epidermal growth factor receptor (EGFR) pathway in EC cells. Knockdown of MUC1 downregulated the expression of EGFR, and further suppressed EGFR-dependent proliferation, growth, and survival. Additionally, MUC1 knockout cells were more sensitive to lapatinib, an EGFR inhibitor [72]. Integrins showed reduced homogenous adhesion in tumor cells and they are the carriers of N-glycans, which plays a role in mutual recognition and adhesion between cells and the extracellular matrix. Tunicamycin is an N-linked glycosylation inhibitor that reduces MUC1 concentration and inhibits MUC1 glycosylation, and the downregulation of MUC1 increases the expression of α2ß1 integrin to promote cell adhesion [60]. CD44 is a complex transmembrane adhesion glycoprotein, and the adhesion between tumor cells and the host cell’s matrix promotes invasion and metastasis. The glycosylation inhibitor tunicamycin is known to inhibit the glycosylation of CD44 and inhibit the metastatic ability of OC cells [73, 74]. Moreover, the glycosylation modification of CD44 induced by transfection of α1,2-Fuc-T, was reported to enhance cell motility and tumorigenicity in rat carcinoma cells, suggesting similar effects in EC [75, 76].

Glycosylation modification affects the invasion and migration of tumor cells not only through the connection between the extracellular matrix and transmembrane proteins, but also through the regulation of metastasis-related molecular signaling pathways. N-Acetylgalactosaminyltransferase2 (GALNT2) is an enzyme that regulates the initial steps of O-glycosylation of mucin and regulates the malignancy of various cancers. It promotes the malignant characteristics of glioma by regulating the O-glycosylation and phosphorylation of EGFR, further modulating the PI3K/Akt/mTOR axis [77, 78]. Proteomics analysis also showed that GALNT2 was highly expressed in the endometrial hyperplasia group, and was closely related to the activation of the EGFR/Akt/ERK pathway [79]. Not only N-glycosylation and O-glycosylation, but also fucosylation and sialylation play an important role in tumor metastasis. FUT8 catalyzes the addition of fucose unit to the GlcNAc at the end of N-glycans to form core fucosylation, which promotes tumor invasion and migration by regulating downstream pathways, such as TGF-β, EGFR, and Wnt/β-catenin. Other studies reported similar findings in breast cancer [80], small cell lung cancer [81], and hepatocellular carcinoma [82]. Radhakrishnan et al. recently found that the aberrant expression of immature truncated O-glycans played a role in the early onset of cancer, wherein they promoted tumorigenesis by disrupting the basement membrane adhesion and increasing cancer cell proliferation [83]. sTn neo- or over-expression prevents cancer cell growth and adhesion to promote metastasis [28]. Increased levels of sialylated glycans were shown to upregulate the expression of tumor-associated antigens and increase cell detachment through electrostatic repulsion of the negative charges [84]. sTn also inhibits the recognition of cancer cells by the immune cells by preventing the mutual recognition of cell–cell or cell–matrix substances, such as selectins, siglecs, and galectins, thereby protecting the invasion and metastasis ability of tumor cells. The expression level of the T antigen is higher in breast cancer cell lines with higher metastatic ability. Treatment of cancer cells with the synthetic T antigen antagonist, lactulose-L-leucine, was found to decrease cancer cell adhesion [85], further verifying the crucial role of sialic acid glycosylation in tumor metastasis.

### Glycosylation with sex hormone imbalance

Endometrial cancer is a hormone-related malignancy, whose pathogenesis is related to several hormone receptors. O- and N-glycosylated modifications are considered important ways to regulate hormone activity. O-linked glycosylation and N-linked glycosylation play roles in signal transduction, in receptor binding regulation and in  glycoprotein hormones bioactivity alteration [86]. An estimated 40% of EC cases are related to obesity, due to increased conversion of androstenedione into estrone by the excess of adipose tissue, which exposes the endometrium to continuously high levels of estrogens. Furthermore, type II diabetes and insulin resistance are also known to be risk factors for Type I EC. Hyperinsulinemia and insulin resistance affect the level of sex hormones, promoting the onset and development of EC [87]. Fasting insulin levels, insulin resistance index, follicle-stimulating hormone, luteinizing hormone, and estrogen are the family members of heterodimeric glycoprotein hormones, all of which participate in the development of EC. Studies also revealed that people with higher levels of insulin resistance index, fasting insulin level, and estrogen are more susceptible to EC [88]. It has also been shown that the glycosylation of reproductive hormones is associated with tumorigenesis [89]. Recently, several groups had reported that human chorionic gonadotrophin-β promoted tumor development and progression [90]. Hyperglycosylated human chorionic gonadotrophin and human chorionic gonadotrophin-β had similar effects on the apoptosis of endometrial adenocarcinoma cells [91]. Therefore, the glycosylation of human chorionic gonadotrophin may be involved in the onset and development of EC [92]. Steroid 5 alpha-reductase 3, a highly expressed protein in human hepatocellular carcinoma and cervical cancer, plays a role in the earliest steps of N-linked glycosylation and steroid hormone formation, which may further help us in understanding the role of hormone glycosylation in EC [93].

### Glycosylation modification with metabolism

Glucose metabolism is closely related to tumorigenesis and development [94]. Glucose metabolism affects glycosylase and further regulates the glycosylation modifications of protein and its biological functions [95]. 2-Deoxy-D-glucose, an inhibitor that targets glucose metabolism, inhibits the synthesis of N-glycosylation and promotes the apoptosis of tumor cells. Its combination with radiotherapy has synergistic anti-tumor effects [96]. As mentioned earlier, one of the pathogenic characteristics of EC is the increase in glucose metabolism [97]. Abnormal glucose metabolism affects the hexosamine biosynthesis pathway flux, which in turn affects processes such as O-glycosylation, and leads to cellular dysfunction, for example, subjecting EC cell lines to hyperglycemic conditions elevated the activities of the Wnt/β-catenin pathway [77]. Glucose metabolism disorders in diabetic patients were associated with an increased occurrence of EC [97]. Glucose metabolism indicators, such as the body mass index, waist-hip ratio, and insulin resistance index, are all associated with the occurrence of EC [98]. 80% of EC patients are estrogen-dependent type I, and are relatively young patients with accompanying metabolic syndromes, such as obesity, diabetes, hyperinsulinemia, and insulin resistance, further supporting the concept that glucose metabolic disorders promote the occurrence and development of EC [99].

Lipid metabolism disorders are also one of the high-risk factors for EC [94]. Apolipoprotein E (ApoE), an O-glycosylated glycoprotein and part of the high-density lipoprotein, showed antioxidant, anti-inflammatory, and anti-atherogenic properties [100]. The expression of ApoE was found to be altered in gynecological pathologies, such as breast cancer [101], choriocarcinoma [102], and endometrial adenocarcinoma [103], and OC [104]. Studies have reported that the content of ApoE in poorly differentiated EC is 13.1 and 9.7 times higher than that in moderately differentiated and well-differentiated EC, respectively [103]. The structure and degree of glycosylation of ApoE at different positions are different. For example, the degree of C-terminal glycosylation of ApoE in cerebrospinal fluid is elevated [105, 106]. It further affects metabolism by promoting the uptake of cholesterol and high-density lipoprotein [107], and remodeling the tumor microenvironment through extracellular matrix, further increasing the occurrence and development of tumors [108], which may be considered a differentiated factor in gynecological cancers [109].

### Glycosylation and immune modulation

In the humoral immune system, almost all of the immunoglobulins and the complement components are glycosylated [110], suggesting that glycosylation plays an indispensable role in the innate and adaptive immune response. Glycan-binding receptors, also known as lectins, are present in the immune cells and participate in tumor invasion, metastasis, and immune escape [13]. The diversity in glycosylation modifications of proteins generates a range of different cancer-associated epitopes [111, 112]. The epitopes change as a result of the altered glycosylation patterns may be unique to cancer cells, and a multitude of monoclonal antibodies to these epitopes have been reported [113]. At the same time, the corresponding antibodies could also undergo glycosylation modifications to exert different biological effects. The glycosylation modification sites at the Fc end of the antibodies are usually the binding sites of Fc receptors and C1q [114]. The changes in glycosylation could increase the binding of Fc receptors and C1q to the antibody, thereby increasing their antibody-dependent cell-mediated cytotoxicity and complement-dependent cytotoxicity activity toward the tumor cells [115, 116]. Moreover, researchers found that estradiol treatment elevated the levels of glycosylated epitopes of complement C3 in rat endometrial adenocarcinoma cell lines. Recent studies had demonstrated that sialoglycan-siglec glyco-immune checkpoint interacted with dendritic cells, inducing antibody-dependent cellular cytotoxicity [117, 118]. Siglec-9 inhibits T cell activation by modulating signaling of the T cell receptor [119]. It has also been reported that the increased sialylation of mucin-associated carbohydrates produced by cancer cells caused an asynchronous change in the expression of cyclooxygenase (COX)-2 [120]. The overexpression of Tn and sTn antigens were significantly associated with COX-2 overexpression, which in turn reduced the infiltration of CD8 + T cells and suppressed the host-immune function [120]. Therefore, glycosylation could affect the cytotoxic ability of the immune cells and the expression level of complement factors, which could promote the occurrence and development of EC.

## Clinical application of glycosylation in endometrial cancer

### Characterization of glycan biomarkers in endometrial cancer

The Cancer Genome Atlas proposed the classification of EC into four subtypes according to the types of gene mutation, including hyper-mutated DNA polymerase ε (POLE), microsatellite-instability high (MSI-H), copy-number low, and copy-number high [121]. Two molecular classification schemes, which are the Translational Research in Post-Operative Radiation Therapy in EC (TransPORTEC) molecular classification system [122] and the Proactive Molecular Risk Classifier for EC (ProMisE) [123], were established as the molecular tests for risk stratification. TransPORTEC and ProMisE systems stratified the risk according to the patients’ abnormality in the POLE and p53 genes, both of which showed the potential to be implemented as the standard practice for risk stratification of EC patients. However, both of them are still underdeveloped and need to be further confirmed and validated for their potential clinical relevance [124].

The above-mentioned genetic classification is complex, with the development of glycosylomics technology, glycoprotein biomarkers that carry certain specific glycans are showing increasing clinical potential [125]. Although the diagnosis and treatment of EC have now shifted from histological typing to molecular typing, given the extensiveness of glycosylation modifications, even small changes in glycosylation could contribute to the occurrence and development of tumors. Stratifying the risk helps with the early diagnosis of cancer and improves patient prognosis [123]. A series of glycoproteins used for the detection and monitoring of EC are enlisted in Table 2.

**Table2:** Glycosylation biomarkers in endometrial cancer

Biomarker	Study type	Prognosis	Sample type	Treatment	Patients cohort	Methodology	Results	Reference
CD147	Single institution	Yes	FFPE	Standard treatment (surgery and chemotherapy)	Normal endometrium (*N* = 20), Endometrial hyperplasia (*N *= 10), Adenocarcinoma and Carcinosarcoma (*N* = 134)	IHC	The low expression of EMMPRIN may be a predictor of a good prognosis in patients with EC	[126]
CA153	Single institution	Yes	Serum	–	Endometrial cancer (*N* = 250) Healthy control (*N* = 5848)	ELISA	The serum content of CA153 increased in patients with EC	[31]
sTn	Single institution	Yes	FFPE	Standard treatment (surgery and chemotherapy) special histology underwent external radiotherapy	Endometrial cancer (*N* = 70)	IHC	Strong expression of an sTn antigen associated with COX-2 induction and CD8 T cell immunosuppression and poor prognosis	[120]
Gd and GdA	Single institution	Yes	FFPE	Standard treatment (surgery and chemotherapy)	Endometrial cancer (*N* = 292)	IHC	High Gd is associated with a better survival rate, but highly positive GdA has a poor prognosis in patients with EC	[127]
UPAR	Single institution	Yes	FFPE	–	Endometrial cancer (*N* = 58) and normal (N = 7)	IHC	increasing UPAR protein expression is associated with an increased recurrence and mortality	[128]
C2GnT1	Single institution	Yes	FFPE	–	Endometrial cancer (*N* = 84)	IHC	C2GnT1 is an important indicator of poor prognosis of EC patients	[129]
GALNT2	Single institution	No	FFPE and serum	–	Endometrial hyperplasia (*N* = 32) and normal (*N* = 30), Endometrial cancer (*N* = 30)	IHC/ELISA	The expression of GALNT2 was down-regulated in patients with endometrial hyperplasia and EC	[79]
GalNAc-T6	Single institution	Yes	FFPE	Standard treatment includes surgery and chemotherapy	Endometrial cancer (*N* = 218)	IHC	The elevated level expression of GalNAc-T6 improves the survival rate of patients	[130]

### Glycoconjugated chemotherapy drugs and targeted cancer therapy

At present, anti-tumor drugs are mainly chemotherapeutic agents, which have limited specificity and cause substantial toxicity. However, glycosylated drugs have shown reduced drug toxicity [131]. Paclitaxel, for example, conjugated with monosaccharides has been reported to show promising anti-cancer effects [132]. Adriamycin conjugated with 2-amino-2-Deoxy-D-glucose and succinic acid had superior anti-cancer efficacy by targeting glucose transporter 1 than free adriamycin [133]. Similarly, the clinical application of geldanamycin (GA), an HSP90 inhibitor, is limited due to its strong toxicity, but the galactose and lactose modified GA was reduced by 40 times as compared to the Glucose-GA [134]. Besides, other studies have reported the antitumor effects of drugs modified by glycosylation, such as azomycin, ketoprofen, cadalene, docetaxel, chlorambucil, etc. [135–138]. Protein tyrosine kinases are a class of kinases that catalyze the transfer of phosphate groups on ATP to protein tyrosine residues. The function of tyrosine kinases is closely related to the occurrence, invasion, and metastasis of malignant tumors [139]. Tyrosine kinase inhibitors (TKIs), such as mTOR inhibitors, EGFR inhibitors, human epidermal growth factor receptor 2 inhibitors, and anti-angiogenic drugs, have shown promising clinical application in cancer patients. However, due to low patient response rates, the above TKIs are not clinically applicable in EC [140]. Therapeutic resistance to TKIs may develop through parallel or bypass mechanisms. It is worth noting that receptor protein tyrosine kinases and other highly complex cell surface signaling molecules are glycoproteins, which require post-translational modification by N-linked glycans to achieve appropriate confirmation, function, and distribution into specific cellular compartments [141]. A previous study performed sensitivity screening of 94 lung cancer cell lines against NGI-1, the targeted inhibitor of oligo-saccharyl-transferase (OST), and reported that mutant EGFR was more sensitive to the inhibitor, and OST inhibition caused cell cycle arrest and also inhibited the expression of other EGFR co-expressed receptors, such as the mesenchymal–epithelial transition factor, thereby inhibiting the growth of tumor cells [142]. Furthermore, OST inhibition in combination with radiation or cytotoxic chemotherapy showed synergistic antitumor effects in glioma [143]. FUT8 modifies the activities of both the hepatocyte growth factor receptor and EGFR and affects tumor growth and invasion. Studies have reported an enhanced therapeutic effect of temozolomide in glioblastoma cells upon suppressing FUT8 expression or using the fucosylation inhibitor 2F-peracetyl-fucose [144]. Besides, the expression of EFGR in head and neck squamous cell carcinoma patients is up-regulated, but the clinical response rate of EGFR monoclonal antibody, cetuximab, is less than 20%. The expression of the tumor-related immune antigen PD-L2 was up-regulated in head and neck squamous cell carcinoma, and FUT8, as a transcriptional target of STAT3, played a key role in the glycosylation of PD-L2. The study showed that inhibiting PD-L2 binding to FUT8, or using Stattic to inhibit STAT3, improved the response to cetuximab [145].

### Glycan-based nano-therapies and cancer therapeutics

There are abundant polysaccharides in nature, such as chitosan, dextran, hyaluronic acid, chondroitin sulfate, and heparin, all of which have low toxicity, low immunogenicity, and are easy to be modified by physical or chemical means, enabling the rapid development of polysaccharide encapsulated drugs for cancer therapy. Such drugs may not just be chemotherapeutic drugs but also drugs enabling gene therapy and immunotherapy [146]. Drug-loaded nanoparticles generally improve the therapeutic effects by targeting specific receptors on the surface of tumor cells, including overexpressed antibody fragments [147], carbohydrates [148], peptides [149], and proteins [150]. Meanwhile, nanomaterials are highly permeable due to the enhanced permeability and retention effect [151]. Cisplatin has limited application in metastatic tumors due to its high toxicity and non-targeted delivery. Benefiting from the properties of polymeric nanogels, cisplatin encapsulated within polymeric nano-gels coated with TKH2 mAb targeting the sTn antigen was reported to have synergistic anti-cancer effects in an orthotopic mouse model of pancreatic cancer [152].

The biosynthetic process of glycosylation modification is complex and involves many vital enzymes [153]. Currently, the research focusing on glycosylase inhibitors is still in progress. Tunicamycin inhibits the formation of dolichol carriers that are necessary for the synthesis of N-glycans, therefore inhibiting the transfer of *N*-acetylglucosamine-1-phosphate to dolichol in the biosynthesis of glycoprotein sugar chains of asparagine [154]. Benzyl*N*-acetyl-·-galactosaminide, a typical O-glycosylation inhibitor, prevents the elongation of O-glycans [60]. Treating Ishikawa cells with Benzyl*N*-acetyl-·-galactosaminide and tunicamycin induced an increase in the adhesion ability of the cells, and reduced the binding of alpha2beta1 integrin and MUC1, inhibiting tumor growth and migration [28]. As sTn can be carried by different proteins as mentioned above, the sialylation modification of proteins may cause organ and tumor-specific reactions [155]. The sTn modified protein was shown to have a tumor-promoting effect [28]. The study reported sialic acid levels to be elevated during cancer development [156]. Of note, the well-known liver cancer marker alpha-fetoprotein, the prostate-specific antigen of prostate cancer, and thyroglobulin of thyroid cancer are all sialylated glycoproteins [157]. Changes in the expression of sialylase during sialylation are closely related to breast tumor stage and prognosis [158]. Therefore, targeting tumor sialylation has strong therapeutic prospects for EC, just as previous review have mentioned [159].

### The application of glycans in the immunotherapy of endometrial cancer

Immunotherapy strategies based on glycosylation modifications are mainly divided into three categories, which are glycosyltransferase inhibitors, antibody-based immunotherapies, and vaccines against glycosylated antigens [160]. Some of the mAbs targeting glycosylation-related tumor-associated epitopes are specific for glycolipids, such as gangliosides (GM2, GM3, GD2, and GD3), while others bind to the carbohydrate haptens present on both glycolipids and glycoproteins, including Le^x^ /Le^y^ and SLe^x^ /SLe^a^ glycan hapten structures [161]. Glycoside-specific targeting of proteins had fewer reduced off-target effects and improved anti-tumor specificity [162]. Tumor-associated carbohydrate antigens, which are carbohydrates linked to immunologically active proteins, have been considered the principal targets for therapeutic anti-cancer vaccines [163, 164]. Examples include vaccines targeting the mucin-related antigens for suppression of breast cancer, the gangliosides GM2 and GD3 for treatment of melanoma, and the glycosphingolipid Globo-H for prostate cancer treatment [165, 166]. Dendritic cells are the core components of anti-tumor immunity, reporting the real-time dynamics through antigen cross-presentation to T cells, which possess cancer cell killing abilities. Cancer vaccines and immunotherapies are greatly compromised if the tumor-associated dendritic cells are defective in antigen cross-presentation. Therefore, stimulating dendritic cells to enable sustained antigen cross-presentation, and contribute to the anti-cancer immune response is of great significance. The Mannan–MUC1 fusion protein-mediated stimulation of DCs has been proven to be efficacious in phase I clinical trials [167–169]. For example, MUC1 is the O-glycosylated protein prevalent in breast carcinoma, and the MUC1 lysate-pulsed DCs promote the expression of MUC1-specific CD8 + T cells in breast cancer immunotherapy [170, 171]. The transformation of chimeric antigen receptor T cell immunotherapy (CAR-T) cells enhances their ability to recognize tumor-specific glycosylated antigens, improving their anti-tumor immunotherapeutic effect [161]. Some of the above studies are currently in different stages of clinical trials. However, the majority of the studies on cancer immunotherapy have mainly focused on cancers other than EC, providing a reference for the study of glycosylation targeted immunotherapy in EC.

## Conclusion and future perspectives

Due to the complexity of glycosylation modifications and the unstable structure of the biological samples during the research process, the specificity and integrity of the glycosylation structure may not be fully guaranteed. The structural and functional analysis of glycans needs further development. A typical method is the mass spectrometry fragmentation technology electron transfer/high energy collisional dissociation, which combines high-energy-induced dissociation, and electron transfer dissociation, to effectively improve the identification throughput, coverage depth, and the accuracy of identification of the site of O-glycoproteome [172, 173]. Modified proteome researches with improved sequencing depth and breadth would be helpful for future studies.

In conclusion, the current research on glycosylation has certain limitations. Though few studies have investigated the glycosylation impacts on EC, they are still very valuable and exciting. Any minor modifications to glycosylation may affect the localization and stability of cell surface receptors and their sensitivity to signaling molecules, influencing cellular functions, which may support tumor growth and metastasis, as well as the immune response. Meanwhile, glycoconjugates are a new generation of therapeutic biomarkers, and the glycosidic form of the protein can provide more predictive information. Development of glycosyl-based cancer neo-antigens for cancer vaccines and targeted therapy, such as antibodies against these antigens, and CAR-T therapy has great therapeutic potential. Glycoside-specific targeting protein nanoparticles can limit off-target effects and enhance antitumor specificity. In short, glycosylation provides a new strategy for individualized and comprehensive treatment and also the experimental direction for future research in EC.

## Abbreviations

EC: Endometrial cancer
MS: Mass spectrometry
GlcNAc: *N*-Acetylglucosamine
FUT: Fucosyltransferases
GalNAc: *N*-Acetylgalactosamine
T antigen: Thomsen-friedenreich antigen
Tn: Monosaccharide GalNAc
sT: Sialylated Thomsen-Friedenreich antigen
sTn: Sialylated monosaccharide GalNAc
ELISA: Enzyme-linked immunosorbent assay
IHC: Immunohistochemistry
PCR: Polymerase chain reaction
MALDI-TOF-MS: Matrix-assisted laser desorption ionization time-of-flight mass spectrometry
SELDI-TOF-MS: Surface-enhanced laser desorption/ionization time-of-flight mass spectrometry
HILIC-UPLC: Hydrophilic interaction chromatography
LC-MS/MS: Liquid chromatography–electrospray tandem mass spectrometry
MALDI-MSI: Matrix-assisted laser desorption/ionization mass spectrometry imaging
GalNAc-T6: *N*-Acetylgalactosaminyl transferase 6
OC: Ovarian cancer
MUC1: Mucin1
EGFR: Epidermal growth factor receptor
GALNT2: Acetylgalactosaminyltransferase2
ApoE: Apolipoprotein E
Gd and GdA: Glycodelin and Glycodelin A
COX: Cyclooxygenase
TKI: Tyrosine kinase inhibitors
GA: Geldanamycin
